# Endodontic Management of a Fused Mandibular Second Molar and Paramolar: A Case Report

**Published:** 2010-08-15

**Authors:** Amin Salem Milani

**Affiliations:** 1. Department of Endodontics, Dental School, Tabriz University of Medical Sciences, Tabriz, Iran.

**Keywords:** Fused Teeth, Root Canal Therapy, Tooth Abnormalities

## Abstract

Tooth fusion is a developmental anomaly characterized by the union between the dentin and/or enamel of at least two separately developing teeth. The fusion of posterior teeth is an uncommon occurrence. In this article, we report a rare case of unilateral fusion of a mandibular second molar with a paramolar. Carious exposure mandated endodontic treatment. The unusual morphology and complex root canal system makes diagnosis and treatment difficult. In this case, successful endodontic management was carried out with precise application of hand and rotary techniques.

## INTRODUCTION

Fusion is the union between the dentin and/or enamel of two or more separately-developing teeth [1]. Depending on the stage of tooth development, different degrees of union may occur [2]. Thus, the pulp chambers and root canals may be joined or separated according to the developmental stage at the time of union [3]. Fusion is more prevalent in primary teeth than in permanent dentition [4]. The prevalence of fusion is higher in the anterior region in both dentitions; only a few cases involving molar and premolar teeth have been reported [5][6][7][8][9][10][11]. The occurrence of fusion in permanent posterior teeth is very rare [3][12].

The fusion of two posterior teeth or a posterior tooth and a supernumerary will result in abnormal morphology and excessive width which may create crowding, misalignment, and malocclusions [13]. These teeth are predisposed to caries and periodontal disease, and most of them require extraction [4][8][10]. If endodontic treatment is warranted in these teeth, the procedure will be very complicated due to unusual root canal anatomy [14].

The purpose of this article is to report successful non-surgical endodontic management of a rare case of fusion of mandibular second molar and a supernumerary.

## CASE REPORT

**Figure 1:**
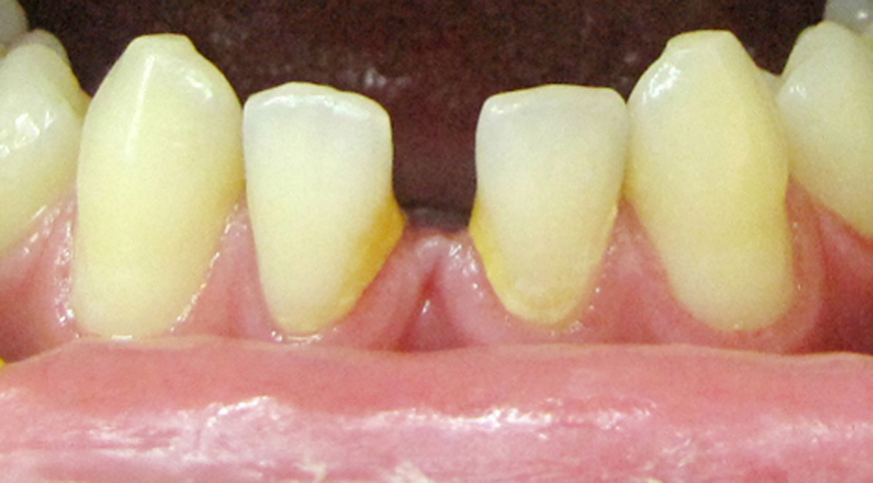
Missing lower central incisors

**Figure 2:**
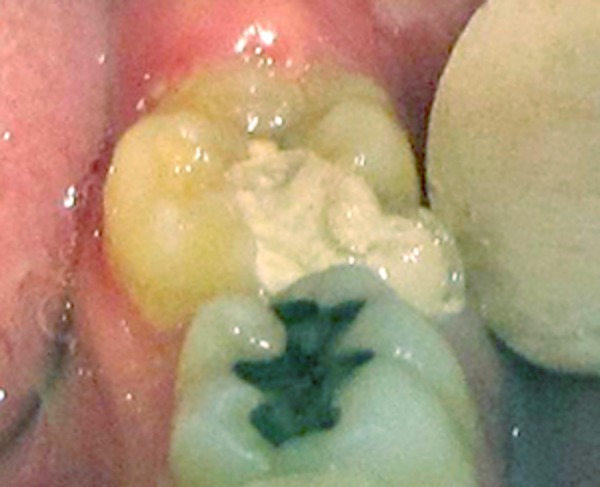
Abnormal morphology of the lower second molar with larger crown suggestive of fusion with another supernumerary tooth

A 22-year-old male patient attended the Tabriz Ostad Shahriar Clinic to have root canal therapy on his left mandibular second molar. Emergency treatment had been performed in another dental clinic. The tooth had been pulpectomized to alleviate the severe pulpal pain. A review of the patient’s medical history revealed no significant findings. Extraoral examination did not show any pathologic finding. Intraoral soft tissues were also normal. Examination of the dentition revealed absence of third molars and lower central incisors (Figure 1). Dental history showed no history of tooth extraction. Clinical examination of left mandibular second molar revealed an abnormal morphology with greater buccolingual and mesiodistal width of the crown than its contralateral counterpart. This finding suggested probable fusion with an adjacent supernumerary tooth (Figure 2).

The tooth was mesiolingually rotated and was not sensitive to percussion or palpation. A shallow occlusogingival groove was present between the two fused teeth on the mesiobuccal aspect. However, the probing depth was within normal limits i.e. between 1-3 mm. The tooth had been temporarily restored following emergency pulpectomy.

**Figure 3:**
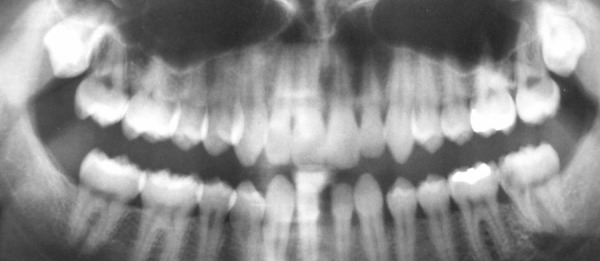
Panoramic radiography taken before emergency visit. Lower incisors and third molars are missing and upper third molars are impacted.

Panoramic radiograph taken before the emergency visit showed bilateral missing lower incisors as well as third molars. The upper third molars were impacted. A large carious lesion was also evident radiographically on mesial surface of the left lower second molar (Figure 3).

**Figure 4:**
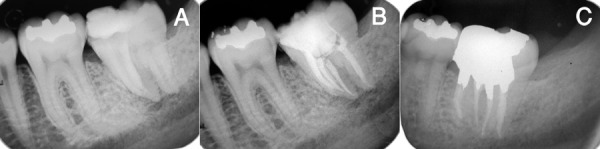
A) Periapical view of the left lower second molar, B) Final periapical radiography, C) Periapical view on one-year follow up

Preoperative periapical radiograph was not available, but the radiograph after emergency visit showed that fusion had occurred at mesiobuccal angle of the tooth. However, the fused teeth had separated roots. Periapical radiolucencies were also evident on the mesial and distal roots of the second molar (Figure 4A).

The tooth was anesthetized, and an access cavity was prepared. The tooth was isolated with rubber dam. The second molar had three separate mesiobuccal (MB), mesiolingual (ML), and distal (D) canals. The paramolar tooth had a single canal located mesiobuccally to the MB canal of the second molar. Working lengths were measured with electronic apex locator (Raypex 5, VDW, Germany) and confirmed by radiography. The single canal of the paramolar was relatively large compared to the other canals with initial file #40. Instrumentation of this canal was performed with Flexofiles (Dentsply, Maillefer, Switzerland) using the step back technique. Other canals were instrumented with protaper rotary file (Dentsply, Maillefer, Switzerland). During instrumentation, the canals were irrigated with 5.25% sodium hypochlorite solution. The canals were dried with paper cones and obturated with Gutta percha (Ariadent, Iran) and AH26 (Dentsply, Konstanz, Germany) sealer using lateral compaction technique (Figure 4B). The tooth was permanently restored with amalgam (Synalloy, Dentoria, France).

At the one-year recall, the patient had no signs or symptoms and radiographic examination revealed resolution of the apical lesion (Figure 4C).

## DISCUSSION

The prevalence of tooth fusion is estimated to be 0.5-2.5% in deciduous dentition [15] much lower than the permanent dentition [10]. The root canal system of fused teeth depends on their developmental stage at the time of union. When fusion occurs at an early stage, the root canal system of the two teeth will merge resulting in a complicated morphology [2][3][4][5][9][10][16][17]. However, fusion in the final stages results in two separate root canal systems.

In this case, the patient attended the clinic after pulpectomy. Therefore, the internal anatomy of pulp chamber could not be assessed. However, the root canal systems were clearly separate. In a similar case reported by Ballal et al., communications between the mesiobuccal canal of the lower second molar and single canal of paramolar-fused root canal system were evident [16].

Fused teeth have a high predisposition to caries and periodontal diseases due to their abnormal anatomy, and if endodontic treatment is needed, the clinician may encounter particular problems [18]. Access cavities can be prepared as two separate coronal entities to preserve as much tooth structure as possible [2][16]. Subsequently, if any communication between the cavities under the dentinal septum is discovered, they can then be merged together.

This also facilitates straight line access into the root canals and so treatment can be performed as one endodontic therapy on the tooth [2][5][6][16][19][20]. However, in one of the cases reported by Tsesis et al., the two cavities were separate and therefore the dentin septum was preserved [2].

Root canal location in fused teeth is an intricate and challenging procedure due to unusual internal anatomy. In this case, four canals were found: three canals were associated with the second molar and a single canal was related to the paramolar.

Placement of rubber dam may sometimes be complicated as a result of the unusual size and shape of the crown. In this case, the beaks of the clamp had to be placed more mesially on the buccal side to establish a four-point contact.

Canal instrumentation of fused teeth is usually another problem. The isthmus between the canals is almost impossible, to clean with the current mechanical instrumentation techniques. Effective irrigation with sodium hypochlorite is very important in eliminating infection and the remaining tissues [2][5][6][16][19][20]. Some researchers believe that even if communication between the root canal systems is initially not clinically discernable, it is recommended that endodontic treatment be performed on these teeth as one entity. In our case, two root canal systems seemed to be separate.

Fortunately, there were no acute signs or symptoms during examination nor any exudates at the time of obturation after initial drainage from the MB and ML canal. The canals could be immediately dried, so the treatment was completed in one session.

## CONCLUSION

Any abnormality in a tooth’s external morphology during examination should direct the clinician to the possibility of abnormal internal anatomy. During the management of fused teeth, the clinician faces multiple problems due to intricate and unpredictable anatomy that requiregreater skill. Endodontic treatment of fused posterior teeth was presented in this case study. At the one year recall, patient had no signs or symptoms and radiographic examination revealed resolution of the lesion. Most of these teeth may be saved with a timely and conservative treatment.
